# Dynamic Up-Regulation of PD-L1 in the Progression of Oral Squamous Cell Carcinoma

**DOI:** 10.3390/ijms242216386

**Published:** 2023-11-16

**Authors:** Sonja Steen, Karl Semmelmayer, Christa Flechtenmacher, Jürgen Hoffmann, Kolja Freier, Dominik Horn, Jochen Hess, Christian Freudlsperger, Julius Moratin

**Affiliations:** 1 Department of Oral and Cranio-Maxillofacial Surgery, Heidelberg University Hospital, 69120 Heidelberg, Germany; sonja.steen@med.uni-heidelberg.de (S.S.); karl.semmelmayer@med.uni-heidelberg.de (K.S.); juergen.hoffmann@med.uni-heidelberg.de (J.H.); christian.freudlsperger@med.uni-heidelberg.de (C.F.); 2 Institute of Pathology, Heidelberg University Hospital, 69120 Heidelberg, Germany; christa.flechtenmacher@med.uni-heidelberg.de; 3 Tissue Bank of the National Center for Tumor Diseases (NCT), 69120 Heidelberg, Germany; 4 Department of Oral and Maxillofacial Surgery, Saarland University Hospital, 66421 Homburg, Germany; kolja.freier@uks.eu (K.F.);; 5 Department of Otorhinolaryngology, Head and Neck Surgery, Heidelberg University Hospital, 69120 Heidelberg, Germany; jochen.hess@med.uni-heidelberg.de; 6 Research Group Molecular Mechanisms of Head and Neck Tumors, German Cancer Research Center (DKFZ), 69120 Heidelberg, Germany

**Keywords:** PD-L1, oral cancer, recurrence, HNSCC, metastases, immune checkpoint inhibitor

## Abstract

The introduction of immune checkpoint inhibition for recurrent and metastatic head and neck cancer has brought a new treatment option for patients suffering from advanced oral cancers without a chance for curation using surgery or radiotherapy. The application of immune checkpoint inhibitors in most cases is based on the expression levels of PD-L1 in the tumor tissue. To date, there is a lack of data on the dynamic regulation of PD-L1 during disease progression. Therefore, this study aimed to evaluate the expression levels of PD-L1 in a large cohort of patients (n = 222) with oral squamous cell carcinoma including primary and recurrent tumors. Semiautomatic digital pathology scoring was used for the assessment of PD-L1 expression levels in primary and recurrent oral squamous cell carcinoma. Survival analysis was performed to evaluate the prognostic significance of the protein expression at different stages of the disease. We found a significant up-regulation of PD-L1 expression from primary disease to recurrent tumors (mean PD-L1 H-scores: primary tumors: 47.1 ± 31.4; recurrent tumors: 103.5 ± 62.8, *p* < 0.001). In several cases, a shift from low PD-L1 expression in primary tumors to high PD-L1 expression in recurrent tumors was identified. Multivariate Cox regression analysis did not reveal a significantly higher risk of death (*p* = 0.078) or recurrence (*p* = 0.926) in patients with higher PD-L1 expression. Our findings indicate that the exclusive analysis of primary tumor tissue prior to the application of checkpoint blockade may lead to the misjudgment of PD-L1 expression in recurrent tumors.

## 1. Introduction

Head and neck squamous cell carcinomas (HNSCC) are a heterogeneous but common malignancy with a worldwide incidence of more than 800,000 cases every year [1]. The disease arises from the mucosal epithelia of the oral cavity, pharynx, and larynx. The therapy of choice primarily depends on tumor stage and tumor localization and comprises surgery, radiotherapy, and systemic therapy. Tumors in the oral cavity are predominantly treated surgically, while radiotherapy is commonly used as adjuvant therapy and for unresectable tumors [2]. Despite multimodal treatment regimens, 5-year survival rates have remained around 40–60% over the past decades [3,4,5,6,7]. Locoregional disease recurrence and metastasis are the main predicting factors for adverse clinical outcomes in affected patients, as prospects for curative treatment are limited [8,9]. Immunotherapy with the programmed death 1 inhibitor pembrolizumab has been established as the first-line therapy for recurrent and metastatic disease and as a monotherapy for programmed death ligand 1 (PD-L1)–positive disease or with platinum plus fluorouracil independent of PD-L1 status [10,11]. Together with nivolumab, pembrolizumab is recommended for second-line treatment of recurrent and metastatic HNSCC after progression on or after platinum-containing therapy [11]. Therefore, immune checkpoint inhibition targeting the PD-1/PD-L1 axis has brought a significant improvement in disease control and long-term outcome for a relevant number of patients [10,12,13,14,15,16].

The up-regulation of PD-L1 has been demonstrated for a variety of malignancies including HNSCC and the expression of PD-L1 in tumor cells has been identified as part of an immune escape mechanism to evade detection by effector cells of the immune system [17,18]. Several authors have described a correlation between high PD-L1 expression with the development of cervical metastases and poor clinical outcome in HNSCC [17,19,20,21]. Furthermore, it has been shown that PD-L1 expression is predictive of treatment response on immune checkpoint inhibition with increased efficacy of therapy in patients with higher PD-L1 expression [15,22].

In a previous study, we demonstrated higher PD-L1 levels in lymph node metastases compared to their corresponding primary tumors [19]. Since the main application of immune checkpoint inhibition is for recurrent or metastatic HNSCC, there is an urgent need to investigate the dynamics of PD-L1 expression during disease progression. Therefore, we analyzed local PD-L1 expression level recurrence and their corresponding primary tumors in a large cohort of surgically treated patients with oral squamous cell carcinomas. In addition, we investigated the resulting impact of PD-L1 expression on survival.

## 2. Results

### 2.1. Patient Cohort

A total number of 222 patients were included in the analysis. Of these, 137 patients (61.7%) were male, and 85 (38.3%) were female. Patient age ranged from 27 to 88 years with a mean age of 64.3 ± 11.1 years. All patients received primary surgical treatment in the Department of Oral and Cranio-Maxillofacial Surgery of the University of Heidelberg between 2010 and 2017. Table 1 provides an overview of the demographic and clinical features of the reported cohort.

### 2.2. Expression of PD-L1 in Primary Tumors

**Figure 1:**
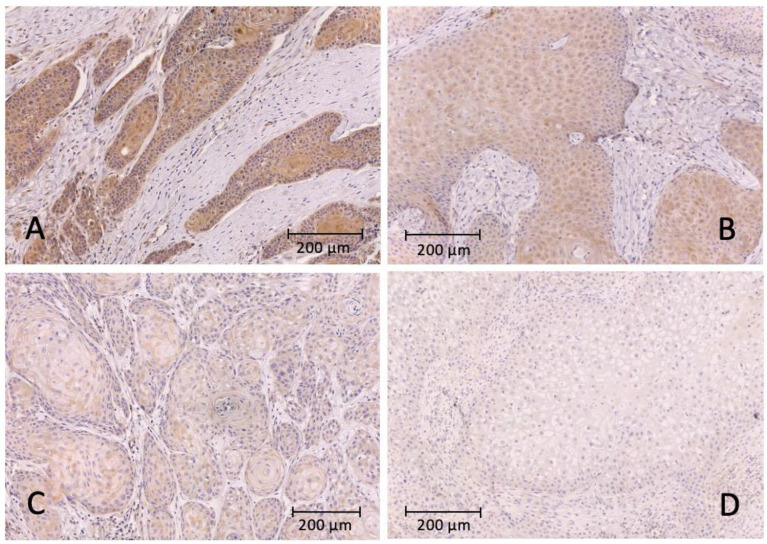
Representative images of immunohistochemical staining with the PD-L1 antibody and hematoxylin as counterstain. (**A**) strongly positive signal of the tumor cells; (**B**) moderately positive intensity of the tumor cells; (**C**) weakly positive intensity of the tumor cells; (**D**) no positive tumor cell staining.

Figure 1 shows tissue samples treated with anti-PD-L1 antibodies with different staining intensities, and overall, 222 primary tumor samples from 222 patients were revised for PD-L1 expression. Two spots per TMA were evaluated. PD-L1 expression could not be evaluated in 28 primary tumors. The mean H-score for PD-L1 in primary OSCC was 47.1 ± 31.4 (Table 2) and did not differ between tumors from male (44.46 ± 29.19) and female (51.02 ± 34.3) patients (*p* = 0.169). In a cross-tabulation analysis with clinicopathological data, we found a significant positive correlation between PD-L1 expression increasing tumor size (*p* = 0.011), and clinical stage (*p* = 0.042). There was no significant correlation between the presence of cervical lymph node metastases (*p* = 0.48), sex (*p* = 0.192) and age (*p* = 0.45). The results of the correlation analysis are shown in Table 3.

### 2.3. Expression of PD-L1 in Recurrent Tumors

Out of 222 patients, 46 experienced disease recurrence. Of these 46 patients, 23 had received adjuvant therapy (radiotherapy: 19; chemoradiotherapy: 3; radioimmunotherapy: 1; see Table 4) before the recurrence. Whole-mount sections of 33 recurrent tumors from 33 patients were available and revised for PD-L1 expression as described. The mean H-Score for PD-L1 expression was 103.53 ± 62.78 and did not differ between male (108.38 ± 66.59) and female (95.03 ± 57.26) patients (*p* = 0.550).

### 2.4. Comparison of PD-L1 Expression in Primary and Recurrent Tumors

**Figure 2:**
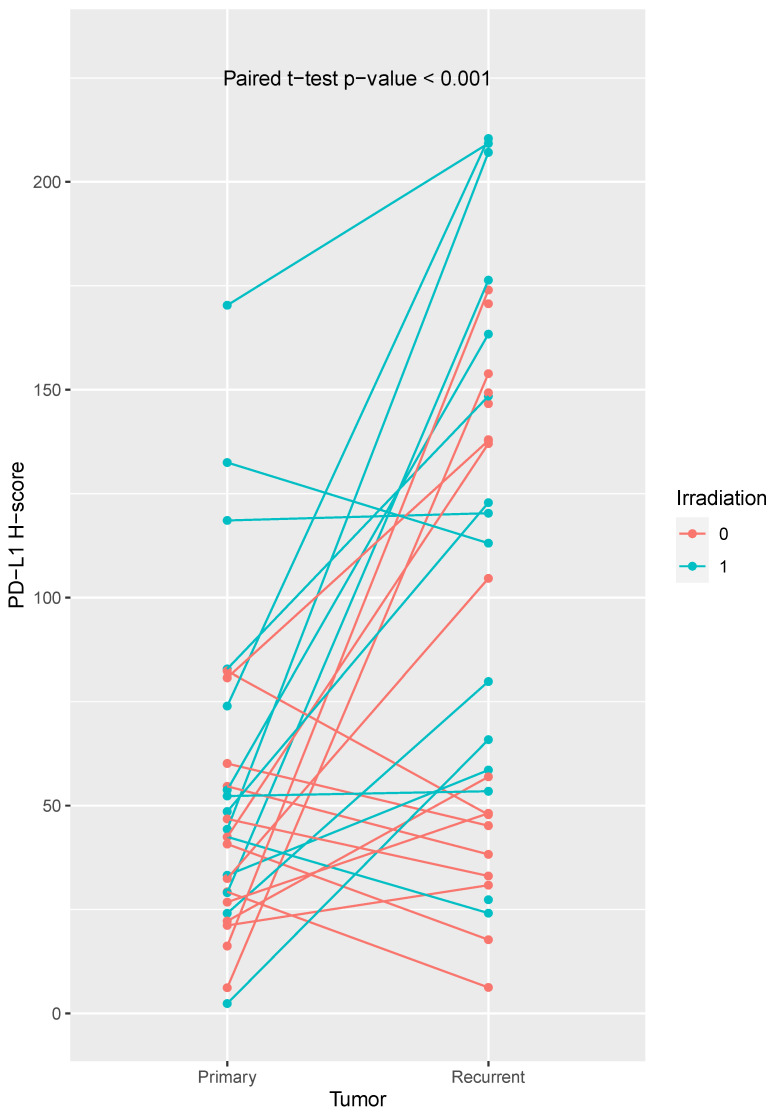
Comparison of PD-L1 expression H-scores of primary and recurrent tumors. We observed a higher PD-L1 expression in recurrent tumors (paired *t*-test, *p*-value < 0.001). Some patients showed a decrease in PD-L1 expression. PD-L1 expression was significantly increased in recurrent tumors from previously irradiated patients. Irradiated patients are shown in turquoise, non-irradiated patients in orange.

We compared PD-L1 the expression in 33 recurrent tumors with their corresponding primary tumors and observed a higher PD-L1 expression in the recurrent tumors (paired sample *t*-test: *p* < 0.001; Figure 2). However, some patients showed a decrease in PD-L1 expression. We did not observe a positive correlation between the PD-L1 expression (H-score) between primary and recurrent tumors (r = 0.19, *p* = 0.33).

**Figure 3:**
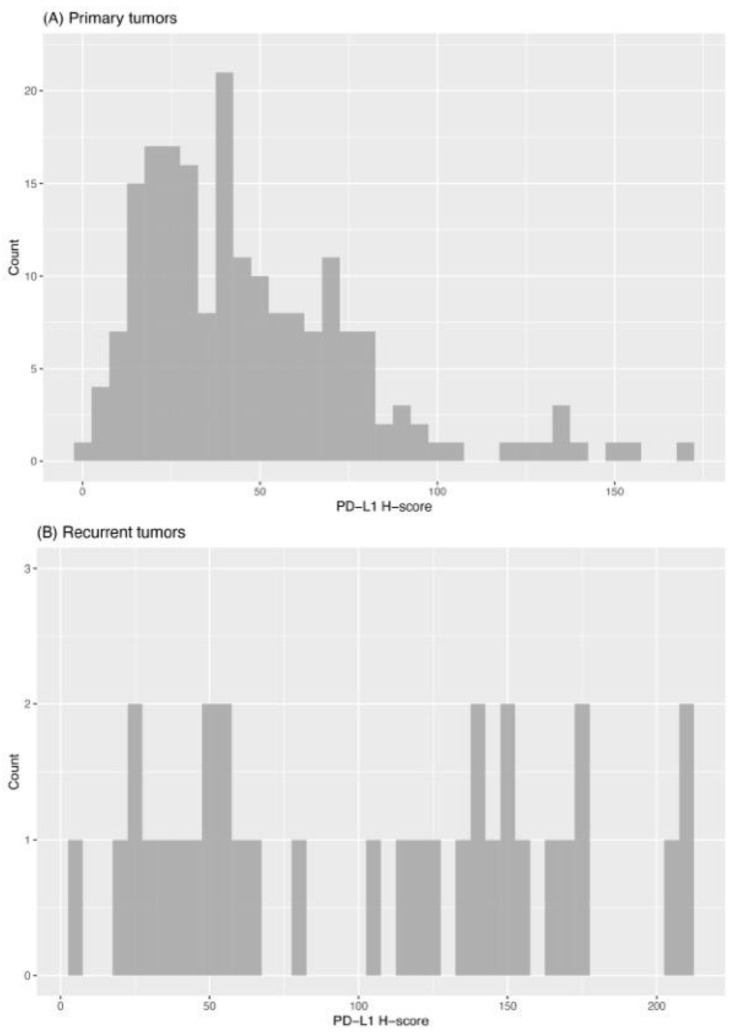
Histogram of PD-L1 expression (H-scores) in primary (**A**) and recurrent (**B**) tumors.

Subsequently, tissue samples were divided into low and high PD-L1 expression groups based on their H-score (low expression group: H-Score 2.4–60; high expression group: H-Score 61–170). We found that primary tumors showed high PD-L1 expression in 27% (53/194), while recurrent tumors showed high PD-L1 expression in 60% (20/33). The frequencies of the PD-L1 expression levels are demonstrated in Figure 3.

The PD-L1 expression in the primary tumors of patients who later experienced recurrence (PD-L1 H-score: 49.68 ± 34.82) did not differ from patients without recurrence (PD-L1 H-score: 46.39 ± 30.53) (*t*-test, *p* = 0.56). the mean values of PD-L1 expression in primary and recurrent tumors are presented in Table 2.

We found a tendency for higher PD-L1 expression in recurrent tumors from non-irradiated patients (PD-L1 H-score primary tumor: 40.15 ± 22.95; recurrent tumor: 73.68 ± 55.74; matched *t*-test, *p*-value = 0.071). Noteably, PD-L1 expression was significantly increased in recurrent tumors from previously irradiated patients (PD-L1 H-score primary tumor: 67.64 ± 47.36; recurrent tumor: 121.28 ± 63.51; matched *t*-test, *p*-value = 0.006).

### 2.5. Overall and Progression Free Survival

In total, 47 patients died during follow-up (21%), while 175 (79%) patients remained alive at the end of follow-up. Overall survival was worse for patients with high PD-L1 expression within the primary tumor (log-rank test, *p* = 0.043). In patients with low PD-L1 expression, the mean overall survival was 69 months (SD = 2.085 months), while patients with high PD-L1 expression survived 59 months (SD = 4.20 months) on average. Regarding progression-free survival, PD-L1 expression had no significant impact.

Univariate analyses of the clinicopathologic characteristics in relation to progression-free and overall survival were performed. High PD-L1 expression (*p* = 0.048), increasing tumor size (*p* = 0.004), cervical lymph node metastasis (*p* < 0.001), and higher UICC stage (*p* < 0.001) were significantly associated with poor overall survival (see Table 5). There was no significant correlation between PD-L1 expression and progression-free survival (*p* = 0.879, see Table 6).

Multivariate Cox regression analysis did not reveal a significantly higher risk of death (*p* = 0.078) or recurrence (*p* = 0.926, see Table 6) in patients with higher PD-L1 expression. However, lymph node metastases and higher UICC stage are associated with a worse overall survival (see Table 5).

## 3. Materials and Methods

### 3.1. Patients and Samples

The cohort comprised 222 patients with primary oral squamous cell carcinoma and 33 matched recurrent tumors (local and regional recurrence), who had a undergone diagnosis and primary surgical treatment in the Department of Oral and Maxillofacial Surgery of the Heidelberg University Hospital between 2010 and 2017. Table 1 provides an overview of the demographic and clinical features of the cohort. All patients received surgical tumor resection, elective or therapeutic uni- or bilateral neck dissection and adjuvant radio- or chemo-radiotherapy in cases of positive lymph node metastases, incomplete tumor resection or the presence of histopathological risk factors (perineural or angiolymphatic tumor infiltration). Written informed consent was obtained from all patients and the study was approved by the Ethics Committee of the Medical Faculty of the University of Heidelberg (Ethic vote: S-360/2011). Clinical and therapeutic follow-up was assessed retrospectively via SAP patient management research (SAP, Walldorf, Germany). This study was conducted in accordance with the Declaration of Helsinki.

### 3.2. Tissue Microarray and Histological Slices

Tissue Microarrays and histological slices were generated by the tissue bank of the National Center for Tumor Diseases (NCT) Heidelberg, Germany, following an established protocol, which has been reported earlier [19,23]. HE-stained slides from the paraffin-embedded tissue samples were reviewed by an experienced pathologist and tumor sections were marked to facilitate the selection of appropriate tissue samples. Tissue cores were extracted from the paraffin blocks using the tissue chip microarray (Beecher Instruments, Sun Prairie, WI, USA) and transferred into recipient blocks, followed by paraffin-embedding to create TMA blocks. Subsequently, sections with a thickness of 2–3 µm were prepared from the TMA blocks for the staining process (Histo Bond, Marienfeld, Germany).

### 3.3. Immunohistochemistry and Digital Pathology Scoring

TMAs of primary tumors and whole-mount slides from recurrent tumors were stained using anti-PD-L1 28-8 (ab205921, Abcam, Cambridge, UK) monoclonal antibody and the DAB Substrate Kit (Abcam, Cambridge, UK) following the manufacturer’s instructions. All immunohistochemical slides and TMAs were scanned with the Ventana DP200 slide scanner (Roche Holding AG, Basel, Switzerland).

The analysis of the obtained stains was performed using QuPath version v0.2.2 [24]. As demonstrated earlier, this approach allowed for the semi-automatic digital quantification of immunohistochemical staining providing reproducible and comparable datasets with a high level of concordance with the scoring results obtained using classical manual scoring [25]. Firstly, the TMA dearrayer function was used to infer the TMA grid, followed by the manual exclusion of invalid samples and staining artefacts. For whole-mount sections, the function of the TMA dearrayer was omitted. Staining vectors were then automatically determined for every TMA slide and whole-mount section individually to ensure meaningful quantification between slides. Tumor cells with staining of the cell membrane were evaluated as positive. We did not observe any difference in PD-L1 expression pattern between primary and recurrent tumor. Next, the positive cell detection function was used to quantify the number of positive cells and by setting an individual threshold for the PD-L1 antibody. For whole-mount sections, individual tissue classification was used to ensure that only positive staining in tumor tissue was considered. The staining intensity was assessed and the staining intensities were classified as “no staining”, “low staining”, “moderate staining”, and “high staining”. Finally, QuPath generated an H-score composed of the percentage of positive cells and the staining intensity. The H-score technically ranges from 0 (all tumor cells negative) to 300 (all tumor cells strongly positive) as described elsewhere [24].

### 3.4. Statistical Analysis

Statistical analysis was performed using Microsoft Excel (Microsoft, Redmond, Washington) and SPSS 27 (SPSS for Windows, SPSS, Chicago, IL, USA). Cross-tabulation and chi-square tests were used to determine the association betweens PD-L1 expression scores and clinical data.

Survival analysis was carried out using the Kaplan-Meier method and log-rank testing was used to determine differences between the groups. Univariate and multivariate Cox regression models were applied to evaluate the impact of PD-L1 expression on overall survival and progression-free survival together with relevant covariates. A *p*-value of less than 0.05 was considered significant.

## 4. Discussion

In the present study, we investigated the expression of PD-L1 in primary and recurrent oral squamous cell carcinoma in a cohort of patients who underwent primary surgical treatment followed by radio(chemo)therapy, if required. The role of immune-modulatory checkpoint mechanisms in oral carcinomas is not yet fully understood. Their regulation in the recurrence of disease has not yet been clarified, and the data regarding their predictive value are controversial. This study aimed to further characterize the dynamic expression profile of PD-L1 in the disease progression of oral squamous cell carcinoma and assess its role as a prognosticator.

In this study, we found a mean PD-L1 H-score in primary tumors of 47.1 ± 31.4, while the mean PD-L1 H-Score in recurrent tumors was 103.53 ± 62.78. The PD-L1 expression differed between primary tumors and their matched recurrent tumors (paired *t*-test: *p* < 0.001).

We demonstrated a longitudinal heterogeneity of PD-L1 expression with higher expression levels in local and cervical recurrences of oral squamous cell carcinoma compared to their matched primary tumor. Our findings are consistent with a previous assessment of longitudinal PD-L1 expression in head and neck cancer, where a 33–36% temporal heterogeneity of PD-L1 expression has been reported depending on the chosen threshold used for CPS scoring [26]. In this context, we want to emphasize that head and neck cancer summarizes different tumor entities of distinct localizations with relevant tumor heterogeneity and wide disparity regarding their prognosis. Importantly, qualitative differences in PD-L1 expression between oral and oropharyngeal squamous cell carcinoma have been presented [27]. We previously reported a PD-L1 up-regulation in lymph node metastases in the primary setting, although we used a different methodology at that time [19]. These observations regarding a heterogeneous PD-L1 expression in the context of lymph node metastasis have been similarly reported by others [28,29].

In addition, we observed an increased PD-L1 expression in recurrent tumors from irradiated patients, which has also been reported by others [30,31,32]. A recent study investigated the modulation of PD-L1 expression in irradiated head and neck cancer cell lines [33]. Affolter et al. reported a significant and dose-dependent increase in PD-L1 expression levels in irradiated cell lines, which was further enhanced after chemoradiation. The authors hypothesized a co-regulation between ERK1/2 activation and PD-L1 expression as the underlying mode of action in the irradiated tumor cells. Moreover, exosomes derived from irradiated cells demonstrated a protective effect on the unirradiated tumor cells, suppressing apoptosis through downregulation of Caspase 3/7. This study proposes a tumor cell-mediated regulation of PD-L1 in response to platinum-based chemoradiotherapy and clearly demonstrates that chemoradiotherapy can influence immune checkpoint regulation in HNSCC.

Overall, PD-L1 appears to exhibit a dynamic expression in both primary metastasis and secondary recurrence. Therefore, we argue that an adequate biopsy should be taken from the current tumor tissue before initiation of anti-PD-L1 therapy. This should be done independently of PD-L1 expression in the primary tumor to be able to consider PD-L1 expression at the time of the application of immune checkpoint blockade. At present, immune checkpoint blockade is used in R/M disease, but a curative efficacy has not been proven so far [34,35]. Determining PD-L1 expression at relapse could potentially identify more patients suitable for immune checkpoint blockade thereby, improving patients prognosis.

The risk factors or clinical variables, which are associated with PD-L1 expression are still not clear. We found a positive correlation between f PD-L1 expression with increasing tumor size and clinical stage. However, there was no significant correlation with the presence of cervical lymph node metastases, sex, and age. Although, Schneider et al. reported consistent results for sex and age [28], Lin et al. demonstrated a positive correlation between male sex and positive smoking history [20], while others identified female sex as a risk factor for increased PD-L1 expression [36]. Thus, there is no consensus on risk factors within the literature. In addition, we could not detect clinical risk factors for high PD-L1 expression in recurrent tumors.

Overall survival was worse for patients with high PD-L1 expression within the primary tumor (log-rank test, *p* = 0.043). However, we did not detect any effect of PD-L1 expression on overall and progression-free survival in this cohort using multivariate analysis. This may be explained by the positive correlation between PD-L1 expression with tumor size and tumor stage in our cohort and is contrary to our previous observations [19]. However, in this cohort, additional patients were included, and were followed up over a longer period, which might explain the disparities. The predictive value of PD-L1 expression has not been clarified and recently published meta-analyses come to different conclusions [37,38,39]. PD-L1 expression appears to be associated with several clinicopathological factors (e.g., sex, tumor stage), which makes interpretation as a predictor difficult [37,38,39].

As this study examined retrospective data and was conducted at a single center, we cannot exclude the possibility of selection bias. As PD-L1 expression varies within head and neck carcinoma due to tumor heterogeneity [40], a potential weakness of this study is the use of tissue microarrays. In the present study, tissue microarrays of primary tumors were compared with whole-slide sections from the recurrent tumors. This is a weakness of the study design. As both tissue microarrays and whole-slide tissue sections do not represent overall tumor heterogeneity [40], we considered the study to be feasible under the study design. Our results are solely based on IHC; however, previous studies showed a strong association between *CD274* amplification and PD-L1 IHC positivity [26,30].

## 5. Conclusions

Our study shows an increase in PD-L1 expression during disease progression of oral squamous cell carcinoma. This should be considered prior to therapy with checkpoint inhibitors to identify suitable patients.

## Figures and Tables

**Table 1:** Descriptive data regarding the demographic and clinical features of the investigated cohort.

Parameters	Number of Cases (%)
Sex	
Female	85 (38.3)
Male	137 (61.7)
Age	
<65 years	112 (50.5)
>65 years	110 (49.5)
T Stadium	
T1	82 (36.9)
T2	72 (32.4)
T3	8 (3.6)
T4	60 (27.1)
N Stadium	
0	147 (66.2)
1	27 (12.2)
2a	1 (0.5)
2b	28 (12.5)
2c	18 (8.1)
3	1 (0.5)
M Stadium	
0	222 (100)
1	0 (0)
UICC	
1	69 (31.1)
2	44 (19.8)
3	23 (10.4)
4	86 (38.7)
Grading	
1	17 (7.7)
2	153 (68.9)
3	46 (20.7)
Missing	6 (2.7)
Resection margin	
R0	210 (94.6)
R1	10 (4.5)
Rx	2 (0.9)
Localization	
Floor of the mouth	64 (28.8)
Tongue	52 (23.4)
Mandible	70 (31.5)
Maxilla	5 (2.3)
Oropharynx	16 (7.2)
Buccal Plane	14 (6.3)
Lower lip	1 (0.5)
Disease recurrence	
yes	47 (21.2)
no	175 (78.8)
Type of recurrence	
Local recurrence	27 (69.2)
Cervical metastases	11 (28.2)
Distant metastases	1 (2.6)
Adjuvant therapy	
No adjuvant therapy	133 (59.9)
Adjuvant radiotherapy	54 (24.3)
Adjuvant chemoradiotherapy	31 (14.0)
Adjuvant radioimmunotherapy	4 (1.8)

**Table 2:** Overview of PD-L1 H-scores of primary and recurrent tumors.

	Average	Median	Minimum	Maximum	Standard Deviation	N
Primary tumors	47.065	40.175	2.38	170.33	31.398	194
with recurrence	49.676	42.465	2.38	170.33	34.822	40
without recurrence	46.386	39.532	4.16	152.57	30.532	154
Recurrent tumors	103.526	113.08	6.26	210.44	62.779	33

**Table 3:** Clinicopathological data and PD-L1 expression (low vs. high) of primary and recurrent tumors (chi-squared test).

	Low PD-L1	High PD-L1	*p* Value
**Primary tumors**			
Sex			
Female	89	28	0.192
Male	52	25	
Age			
<65 years	69	24	0.650
>65 years	72	29	
pT Stadium			
T1/T2	104	29	0.011
T3/T4	37	24	
pN Stadium			
pN−	98	34	0.476
pN+	43	19	
Stage			
I/II	79	21	0.042
III/IV	62	32	
Grading			
G1	12	3	0.786
G2	96	36	
G3	29	12	
**Recurrent tumors**			
Sex			
Female	5	7	0.840
Male	8	13	
Age			
<65 years	8	9	0.353
>65 years	5	11	
pT Stadium			
T1/T2	11	9	0.023
T3/T4	2	11	
pN Stadium			
pN−	9	4	0.005
pN+	4	16	
Stage			
I/II	8	2	0.002
III/IV	5	18	
Grading			
G1	1	0	0.222
G2	8	17	
G3	3	2	

**Table 4:** Descriptive data regarding adjuvant therapy in patients with tumor recurrence.

Parameter	Number of Cases (%)
No adjuvant therapy	23 (50.0)
Adjuvant radiotherapy	19 (41.3)
Adjuvant chemoradiotherapy	3 (6.5)
Adjuvant radioimmunotherapy	1 (2.2)

**Table 5:** Univariate and multivariate analysis regarding the correlation between clinicopathological characteristics and overall survival.

	Univariate		Multivariate	
Characteristics	HR (95% CI)	*p*-Value	HR (95% CI)	*p*-Value
PD-L1 expression	1.97 (1.01–3.86)	0.048	1.86 (0.93–3.72)	0.078
T Classification	2.34 (1.30–4.21)	0.004	2.27 (0.95–5.46)	0.066
N Classification	3.57 (1.99–6.40)	<0.001	3.93 (1.34–11.50)	0.013
UICC stage	3.13 (1.66–5.87)	<0.001	0.70 (0.19–2.67)	0.603
Age	1.057 (0.6–1.88)	0.849	1.09 (0.54–2.17)	0.813
Sex	0.86 (0.47–1.57)	0.615	0.87 (0.43–1.75)	0.687

**Table 6:** Univariate and multivariate analysis regarding the correlation between clinicopathological characteristics and progression-free survival.

	Univariate		Multivariate	
Characteristics	HR (95% CI)	*p*-Value	HR (95% CI)	*p*-Value
PD-L1 expression	1.06 (0.51–2.19)	0.879	0.97 (0.46–2.03)	0.926
T Classification	1.89 (1.04–3.43)	0.038	2.04 (0.87–4.80)	0.102
N Classification	3.66 (2.04–6.59)	<0.001	4.28 (1.36–13.56)	0.013
UICC stage	2.90 (1.55–5.41)	0.001	0.59 (0.15–2.36)	0.459
Age	0.91 (0.51–1.60)	0.735	1.00 (0.51–1.95)	0.997
Sex	0.72 (0.39–1.13)	0.294	0.90 (0.45–1.78)	0.763

## Data Availability

The data presented in this study are available on request from the corresponding author. The data are not publicly available.
